# 

**Published:** 1915-06-05

**Authors:** 


### CONTENTS.

| | Page |
|---|---|
| **National Service and the Hospitals** ... ... | 197 |
| **What is a Clinical Lecture?** ... ... ... | 198 |
| **Hospital and Institutional News ...** ... ... | 199 |

National Service Indeed—Our "Hospital Sunday" Appeal Number—Another Secretary Killed in Action—Precautions Against Poisonous Bombs—Two Beds Endowed at King Edward VII.'s Hospital, Cardiff—The Chairman of the Belgian Field Hospital Resigns—Tribute to our Stretcher-Bearers in the Field—London and the Belgian Red Cross—Hospital Work and Parliament—Promotion in Mental Hospitals—New Superintendent of Fife Mental Hospital—Suicide in a Naval Hospital—A Dilatory Board and their Medical Officer—Patients' Payments at Bridgwater Hospital—The Root of the Failure and the Way Out—Medical Defence Union and Leicester Insurance Committee—A London Park for Hospital Purposes—Boards of Guardians and Soldiers in Hospital—The Scientific Organisation of Military Medicine—Charity Fêtes in War-Time—Deaths Under Anæsthetics—This Week's Drug Market.

| | Page |
|---|---|
| **The Shortage in Hospital Medical Staffs** ... | 205 |
| The Facts and the Remedies. | |
| **The Sphygmometer and Aortic Regurgitation** | 207 |
| An Opportunity for Valuable Work. | |
| **A Testator's Intention** ... ... ... ... | 208 |
| **Water-Cures in Peace and War** ... ... ... | 208 |
| The Medical Bath in History. | |
| **Epsom College and its Medical Pensioners** ... | 209 |
| The Nature and Claims of its Work. | |

### CONTENTS—(*continued*).

| | PAGE |
|---|---|
| **Round the World** ... ... ... ... ... ... | 210 |
| Fellow-Workers and the Roll of Honour. | |
| **War Work at the London Hospital** ... ... | 211 |
| Staff, Salaries, and Air-Raid Decisions. | |
| **Medical Schools and Medical Protection** ... | 212 |
| THE HOSPITAL'S Criticism Endorsed. | |
| **Gifts and Bequests** ... ... ... ... ... ... | 212 |
| **The Institutional Library** ... ... ... ... | 213 |
| From the Russian Trenches. | |
| Chronicles of an Old Campaigner. | |
| "The Knowledge of an Anatomist and the Experience of a Physician." | |
| **The Book Market** ... ... ... ... ... ... | 214 |
| **National Insurance Act Developments** ... ... | 215 |
| The Decision of Disputes. | |
| The Effect of the War on Administration Expenses of Approved Societies. | |
| **The Editor's Letter-Box** ... ... ... ... | 215 |
| Anomalies and Curiosities of Medical Literature. | |
| **The Professional Classes Relief Fund** ... ... | 216 |
| Dr. Scharlieb on its Maternity Work. | |
| **London Panel Committee** ... ... ... ... | vi |
| Excessive Prescribing: An Administrative Breakdown. | |
| **Personalia** ... ... ... ... ... ... ... | vi |
| **The Institutional Worker** ... ... (Suppl.) | 1 |
| Index System for Minute-Books: Answer to April Question. | |
| Questions for June. | |
| Enquiries and Answers. | |
| Miscellaneous: The "Berkefeld" Filter. | |
| Practical Matters: Cost of Provisions—Air-Space for Bedrooms. | |
| Employment and Training: Infirmary Probationers—Addresses Wanted—Scotch Fever and Poor-Law Hospitals—Troubled by Recruiting. | |

